# Lysosomal modifications in astrocytes exposed to pathological α‐synuclein

**DOI:** 10.1002/alz70855_102076

**Published:** 2025-12-23

**Authors:** Magdalena M. Bolsinger, Bruno Ghirotto, Denise Balta, Raul Moritz, Jan Dobert, Philipp Arnold, Beate Winner, Friederike Zunke

**Affiliations:** ^1^ Friedrich‐Alexander‐University Erlangen‐Nürnberg, Erlangen, Bavaria, Germany

## Abstract

**Background:**

Astrocytes play a pivotal role in the clearance and turnover of α‐synuclein (αS), a protein implicated in the pathogenesis of dementia with Lewy bodies (DLB), Parkinson's disease, and other α‐synucleinopathies. Lysosomes play a central role in degrading pathological αS and other aggregated proteins associated with neurodegeneration. However, despite their critical roles, the mechanisms underlying astrocytic lysosomal responses to αS pathology remain poorly understood.

**Method:**

To elucidate these mechanisms, we analyzed the lysosomal system in primary wild‐type astrocytes (WT), iPSC‐derived astrocytes, and primary astrocytes from an αS‐overexpressing mouse model (MBP29: human αS‐overexpression in oligodendrocytes). We assessed key lysosomal enzymes and membrane proteins linked to αS pathology, including LAMP‐2, LIMP‐2, cathepsins B and L, and β‐glucocerebrosidase (GBA), regarding expression, maturation, localization and functionality utilizing Western blotting, immunofluorescence, lysate and live cell analyses, and mass spectrometry. Additionally, we evaluated the astrocytes’ capacity to internalize and degrade fibrillar αS.

**Result:**

In primary WT astrocytes, exposure to fibrillar αS resulted in lysosomal dysfunction, characterized by decreased GBA and cathepsin L activity, and increased cathepsin B activity. Notably, these changes were reversible upon the removal of αS. hiPSC‐derived astrocytes demonstrated a robust ability to internalize and degrade fibrillar αS, with enhanced lysosomal enzyme activity facilitating complete clearance of αS fibrils. In contrast, MBP29 astrocytes exhibited significant lysosomal impairment when exposed to αS‐overexpressing oligodendrocytes, including reduced levels and activities of lysosomal proteins. However, after a two‐week recovery period without αS exposure, these astrocytes showed restored lysosomal function and reduced αS levels.

**Conclusion:**

In summary, our findings reveal that fibrillar αS induces significant, time‐dependent changes in lysosomal enzyme activity and protein expression in astrocytes across multiple cellular models. We demonstrate that astrocytes possess a remarkable capacity to manage pathological αS from exogenous sources. Moreover, our results highlight the dynamic resilience of astrocytic lysosomes in recovering from αS‐induced dysfunction. These findings emphasize the critical role of lysosomal function in astrocytes and suggest that targeting the astrocytic lysosomal system could enhance the degradation of pathological αS, potentially mitigating neurodegeneration and improving clinical outcomes in DLB and other α‐synucleinopathies.

